# Sub optimal HIV status ascertainment at antenatal clinics and the impact on HIV prevalence estimates: A cross sectional study

**DOI:** 10.1371/journal.pone.0278450

**Published:** 2022-12-01

**Authors:** Fatihiyya Wangara, Janne Estill, Hillary Kipruto, Kara Wools-Kaloustian, Wendy Chege, Griffins Manguro, Olivia Keiser

**Affiliations:** 1 Institute of Global Health, University of Geneva, Geneva, Switzerland; 2 Department of Health Services, County Government of Kwale, Kwale, Kenya; 3 Health Systems & Services, World Health Organization, Harare, Zimbabwe; 4 Department of Medicine, Indiana University, Indianapolis, Indiana, United States of America; 5 National AIDS Control Council, Ministry of Health, Nairobi, Kenya; 6 International Center for Reproductive Health, Mombasa, Kenya; Centers for Disease Control and Prevention, UNITED STATES

## Abstract

**Background:**

While many countries including Kenya transitioned from sentinel surveillance to the use of routine antenatal care (ANC) data to estimate the burden of HIV, countries in Sub Saharan Africa reported several challenges of this transition, including low uptake of HIV testing and sub national / site-level differences in HIV prevalence estimates. In Kenya voluntary HIV testing is offered to all 1^st^ ANC clients. However, some women may decline testing. We aim to predict the HIV positivity (as a proxy of prevalence) at ANC assuming 100% uptake of HIV testing and compare this to the observed positivity.

**Methods:**

Using a cross sectional study design, we examine routine data on HIV testing among all women attending ANC in Kwale County, Kenya, for the period January 2015 to December 2019.We used a generalized estimating equation with binomial distribution to model the observed HIV prevalence as explained by HIV status ascertainment. We then used marginal standardization to predict the HIV prevalence at 100% HIV status ascertainment and make recommendations to improve the utility of ANC routine data for HIV surveillance.

**Results:**

HIV testing at ANC was at 91.3%, slightly above the global target of 90%. If there was 100% HIV status ascertainment at ANC, the HIV prevalence would be 2.7% (95% CI 2.3–3.2). This was 0.3% lower than the observed prevalence. Across the yearly predictions, there was no difference between the observed and predicted values except for 2018 where the HIV prevalence was underestimated with an absolute bias of -0.2 percent. This implies missed opportunities for identifying new HIV infections in the year 2018.

**Conclusions:**

Imperfect HIV status ascertainment at ANC overestimates HIV prevalence among women attending ANC in Kwale County. However, the use of ANC routine data may underestimate the true population prevalence. There is need to address both community level and health facility level barriers to the uptake of ANC services.

## Introduction

HIV prevalence estimates is a key indicator to inform the coverage and effectiveness of HIV prevention measures. For many years, countries have used sentinel surveillance data from prevention of mother to child transmission (PMTCT) or antenatal care (ANC) clinics to estimate the burden and trends of HIV infection [1]. While sentinel surveillance can provide valuable information on the burden of HIV, it is not considered representative of the general population hence may over or underestimate the general population prevalence [2,3]. The Joint United Nations Program on HIV/AIDS (UNAIDS) and the World Health Organization (WHO) proposed transitioning from ANC sentinel surveillance to the use of routine ANC data [1]. The latter has the advantage of expanding the representativeness of the sample while reducing logistical and financial challenges of sentinel surveillance [4]. Additionally, it is fully nested within routine health services thus women can receive a comprehensive package of care including psychosocial support and necessary referrals [1,5]. However, the WHO recommends that selection bias inherent in routine HIV testing at ANC be one of the criteria to assess the utility of routine program data. To limit this bias, it is important that the uptake of HIV testing be high at all ANC sites, at about 90% [1].

Kenya transitioned to the use of ANC routine test (ANC-RT) data for surveillance and aimed to eliminate mother-to-child transmission (eMTCT) of HIV by 2021 [6]. To achieve this, targets of 90% (ANC) attendance, 90% HIV testing among pregnant women, and 90% antiretroviral therapy (ART) use among HIV-positive pregnant women were set to be reached by 2019. The policy prescribes that HIV counseling and testing should be offered to all women at first ANC visit, with the exception of women with a previous HIV positive result [7]. The use of ANC-RT is however not without challenges and continuous data quality assessment is essential [8,9]. Additionally, there isn’t much control on the testing and data collection procedures [10] and bias in HIV status ascertainment and subsequently prevalence may result as women can freely opt out of testing [11].

We examine ANC-RT data for Kwale County from January 2015 to December 2019 to estimate HIV prevalence among women attending ANC at 100% HIV status ascertainment. We further estimate the bias in HIV prevalence as a result of imperfect uptake of HIV testing and make recommendations to improve the utility of ANC-RT for HIV surveillance. Comparing observed HIV prevalence at ANC given the current uptake of HIV testing, modelled prevalence (assuming all eligible women attending ANC receive a HIV test) and HIV prevalence from population based surveys would provide insight into the level of bias routine program data estimates could have.

## Materials and methods

This was a cross sectional study using secondary data, conducted in all the four sub-counties of Kwale County, Kenya. This secondary data analysis was conducted between October to September 2020. The study population was all women attending first ANC visit in all the 124 ANC sites in Kwale County over a 5-year period i.e. January 2015 to December 2019.

### Data sources

We used data from the ‘Kenya Health Information System for Aggregate reporting and analysis (KHIS Aggregate)’. This is the national platform for reporting all health-related data. After manual aggregation from source registers, each health facility submits monthly summary reports for validation and entry in KHIS Aggregate. We based our analysis on annual health facility-level data collected over a 5 year period; January 2015 to December 2019. The outcome variable of interest included health facility level HIV positivity at ANC, herein referred to as HIV prevalence whereas the independent variable was HIV status ascertainment.

### Statistical methods

HIV status ascertainment was calculated as:

$${Ascert}_{it}=\frac{{Total\;ANC}_{it}-{Not\;tested}_{it}}{{Total\;ANC}_{it}}$$ (1)

Where *Ascert*_*it*_ is the proportion of women whose HIV status was ascertained at health facility *i* in year *t*; *Total ANC*_*it*_ is the total number of pregnant women attending first ANC in that year and at the health facility; *Not tested*_*it*_ is the number of women with unknown HIV status for the same year and facility. Thus, *Ascert*_*it*_ included both newly diagnosed and previously known HIV positive women. HIV prevalence, *Pr*_*it*_, at facility *i* and year *t* was then estimated as follows:

$${Pr}_{it}=\frac{{KP}_{it}+{New\;positive}_{it}}{{Total\;ascertained}_{it}}$$ (2)

Where *KP*_*it*_ were women with a previous HIV positive result and *New positive*_*it*_ were those diagnosed as HIV positive at first ANC. The outcome variable of interest being ANC HIV prevalence in each health facility each year over a period of 5 years. Thus, the prevalence of HIV in facility *i* in year 2 would not be independent of the prevalence observed in year 1 in the same facility. Due to this longitudinal nature of the data hence likely dependence between yearly observations, we used a generalized estimating equation as opposed to a generalized linear model (GLM) to model observed HIV prevalence. While a generalized linear mixed model (GLMM) is suitable for longitudinal data, our interest in this study was more on the marginal effect rather than the individual effect i.e. what is the effect of extent of HIV status ascertainment on the observed average population prevalence. A binomial distribution of the form $\left({KP}_{it}+{New\;positive}_{it})∼Binomial\;({Total\;ascertained}_{it,}\;{Pr}_{it}\right)$ was applied. Due to the nonlinear relationship between HIV status ascertainment and HIV prevalence, the former was included in the model as restricted cubic splines with three knots. We then used marginal standardization to predict the HIV prevalence in the event the HIV status of all women attending first ANC was ascertained. An unstructured correlation was assumed. Observed and predicted values were compared across years and sub counties.

Additionally, we investigated whether bias in prevalence arose due to presentation of women who were already known to be HIV positive. We fitted the same regression model as described in the preceding paragraphs, but redefined the variables as follows:

$${Test\;uptake}_{it}=\frac{{Tested}_{it}}{{Total\;ANC}_{it}-{KP}_{it}}$$ (3)

Where *Test uptake*_*it*_ is the proportion of eligible women who were tested for HIV at health facility *i* in year *t*. Subsequently, HIV prevalence among women who had no previous HIV positive result was given by ${Pr2}_{it}=\frac{{New\;positive}_{it}}{{Tested}_{it}}$ and distributed as $\left({New\;positive}_{it})∼Binomial\;({Tested}_{it}{,Pr2}_{it}\right)$.

### Ethics

The study utilized retrospective aggregate level data that was fully anonymized before it was accessed for use in the study. As such, participant consent was not applicable. The study was approved by the Pwani University ethics review committee. Reference; ERC/PhD/010/2021.

## Results

A total of 139,754 pregnant women were enrolled during their first ANC visit across 124 HIV PMTCT sites over the five year period. The yearly attendance was fairly constant with a minimum of 23,509 and a maximum of 29,690 women in 2017 and 2018, respectively. The average HIV status ascertainment was at 91.3%, with 2019 and 2017 recording the least values of 85.6% and 88.7%, respectively. Observed HIV prevalence was at 3.0%, but fluctuated across the study period, from a high of 3.7% in 2017 to a low of 2.5% in 2018. HIV status ascertainment differed across the four sub counties, at 87.7%, 88.7%, 92.5% and 96.4% in Matuga, Lungalunga, Kinango and Msambweni, respectively. The observed HIV prevalence was highest in Msambweni Sub County (5.7%) and lowest in Kinango Sub County (1.6%).

Excluding known HIV positive women, 137,724 pregnant women eligible for HIV testing attended their first ANC visit within the County. The average HIV testing uptake was at 91.1%, with 2019 and 2017 recording the least values of 85.3% and 88.6%. Observed HIV prevalence was at 1.4%. Similarly, prevalence was highest in the year 2017 (2.3%) and lowest in 2018 (0.9%). Sub county trends were similar, with Msambweni recording a HIV prevalence of 3.1%. Table 1 summarizes the HIV prevalence trends across time and region.

**Table 1:** HIV prevalence estimates by year and sub county.

**HIV prevalence estimates by year**								
Year	**Model 1: includes women with previous HIV +ve results**				**Model 2: excludes women with previous HIV+ve results**			
	HIV testing uptake (%)		HIV prevalence (%)		HIV testing uptake (%)		HIV prevalence (%)	
	Mean	Median (IQR)	Observed (95% CI)	Predicted (95% CI)	Mean	Median (IQR)	Observed (95% CI)	Predicted (95% CI)
2015	97.2	99.5 (90.6–100)	2.9 (2.2–3.6)	2.7 (2.2–3.1)	97.2	99.5 (90.6–100)	1.5 (1.2–2.8)	1.1 (0.7–1.4)
2016	92.2	94.5 (82.7–100)	3.1 (2.4–3.8)	2.7 (2.2–3.1)	92.0	94.6 (82.5–100)	1.6 (1.1–2.1)	1.0 (0.7–1.4)
2017	88.7	95.2 (81.9–100)	3.7 (1.5–5.9)	2.8 (2.4–3.3)	88.6	95.2 (81.8–100)	2.3 (0.1–4.4)	1.1 (0.8–1.4)
2018	92.1	99.9 89.9–100)	2.5 (1.9–3.0)	2.7 (2.3–3.2)	91.9	99.9 (89.3–100)	0.9 (0.7–1.1)	1.1 (0.8–1.4)
2019	85.6	92.7 (80.5–100)	2.9 (2.2–3.7)	2.7 (2.3–3.1)	85.3	92.7 (80.5–100)	1.1 (0.7–1.5)	1.1 (0.7–1.3)
2015–19	91.3	96.3 (84–100)	3.0 (2.5–3.5)	2.7 (2.3–3.2)	91.1	96.3 (84–100	1.4 (1.0–1.8)	1.1 (0.8–1.4)
**HIV prevalence estimates by Sub County**								
Sub County	Mean	Median (IQR)	Observed (95% CI)	Predicted (95% CI)	Mean	Median (IQR)	Observed (95% CI)	Predicted (95% CI)
Kinango	92.5	96.5 (86.6–100)	1.6 (1.3–1.9)	1.4 (0.9–2.0)	92.4	96.5 86.4–100)	0.6 (0.5–0.8)	0.4 (0.2–0.7)
Lungalunga	88.7	93.7 (81.4–100)	2.5 (1.9–3.0)	2.2 (1.4–3.1)	88.6	93.6 (81.0–100)	1.3 (0.8–1.9)	1.0 (0.3–1.8)
Matuga	87.7	92.2 (77.6–100)	3.3 (2.6–3.9)	2.9 (2.0–3.9)	87.5	92.0 (76.9–100)	1.3 (1.0–1.6)	1.0 (0.4–1.7)
Msambweni	96.4	100 (94.1–100)	5.7 (3.3–8.1)	5.4 (3.5–7.3)	96.4	100 (93.9–100)	3.1 (0.8–5.4)	2.4 (1.5–3.3)

### Predictions of HIV prevalence at 100% status ascertainment

**Fig 1:**
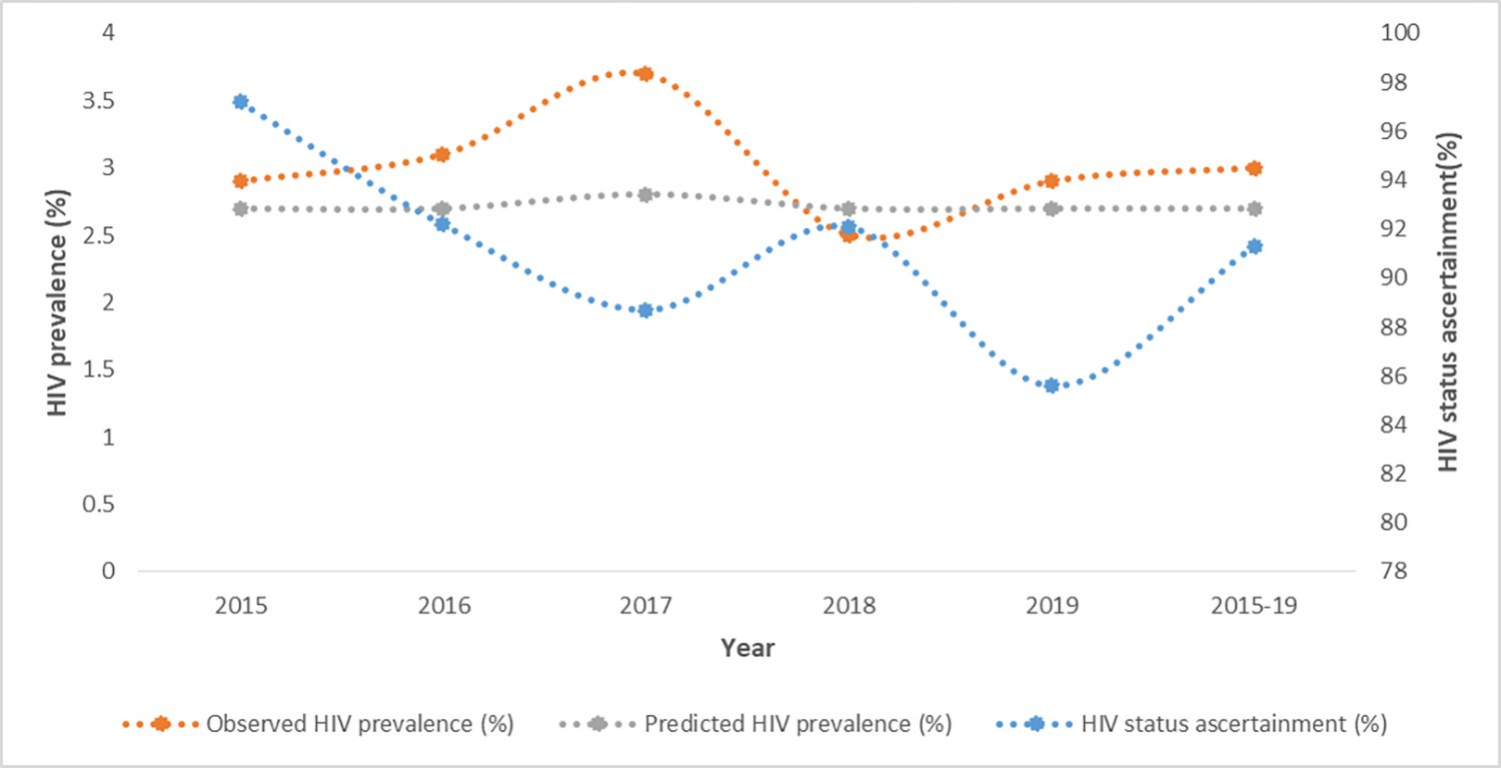
Trends of HIV status ascertainment and HIV prevalence.

**Fig 2:**
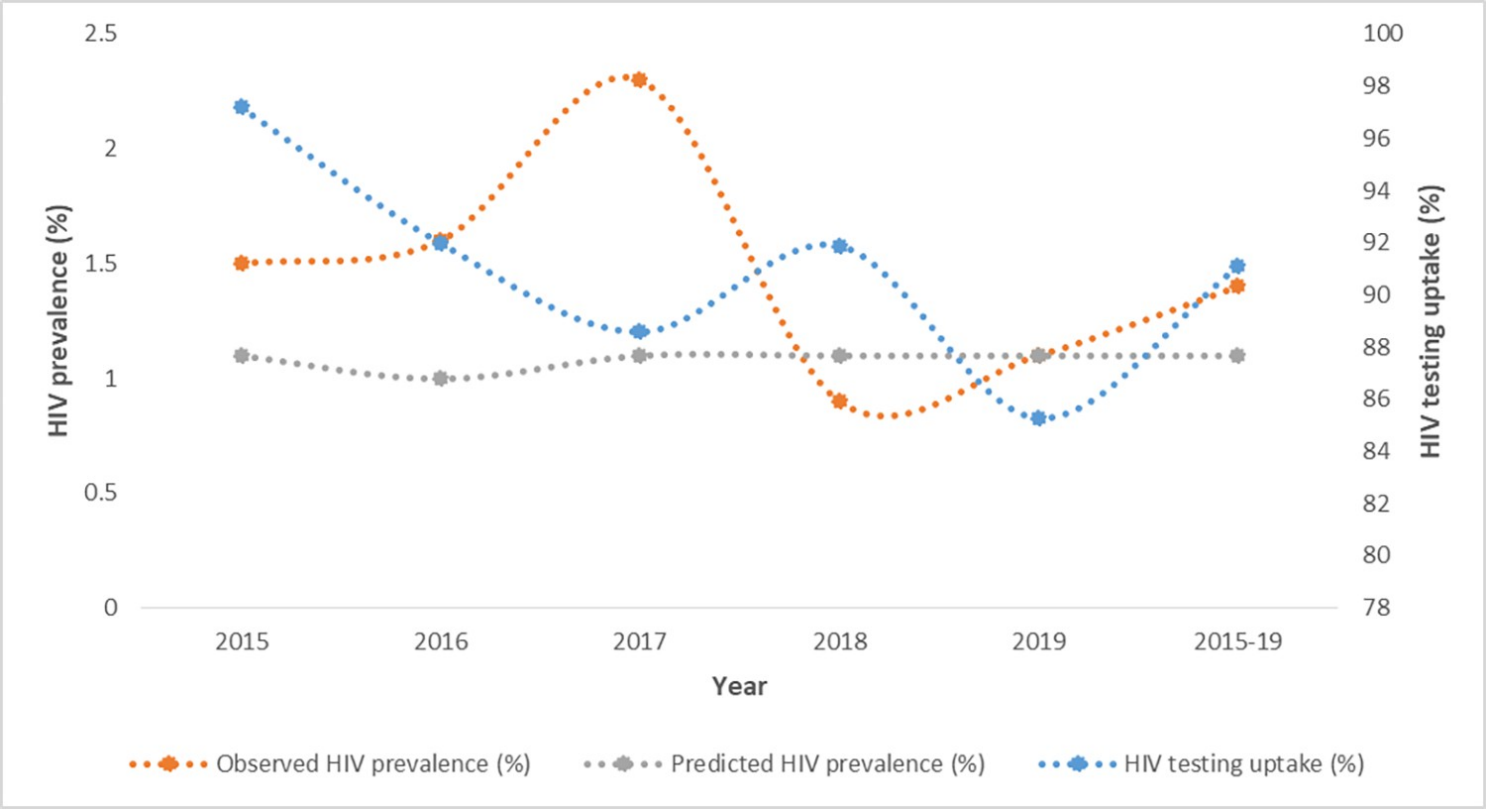
Trends of HIV testing and HIV prevalence.

Two models were fit; one estimating HIV prevalence among all women attending their first ANC visit and the other estimating HIV prevalence among only the women attending first ANC who were eligible for testing. The predicted prevalence was fairly constant across the study period as illustrated in Figs 1 and 2.

Uptake of HIV testing and observed prevalence did not present an obvious trend, with both measurements fluctuating over time.While the highest mean prevalence for both groups was recorded in 2017, alongside low uptake of HIV testing, the differences across the years was not statistically significant.

The model suggests that at 100% HIV status ascertainment, the prevalence would be 0.3 percent lower than the observed. Across the yearly predictions, there was no difference between the observed and predicted values except for 2018 where the HIV prevalence was underestimated with an absolute bias of -0.2 percent. Comparison between observed and predicted prevalence at perfect testing is summarized in Table 1. Model 1 suggests that observed and predicted HIV prevalence did not differ by region. However, in model 2, the observed HIV prevalence was higher by 0.2 and 0.3 percent in Kinango and Lungalunga sub counties respectively.

**Fig 3:**
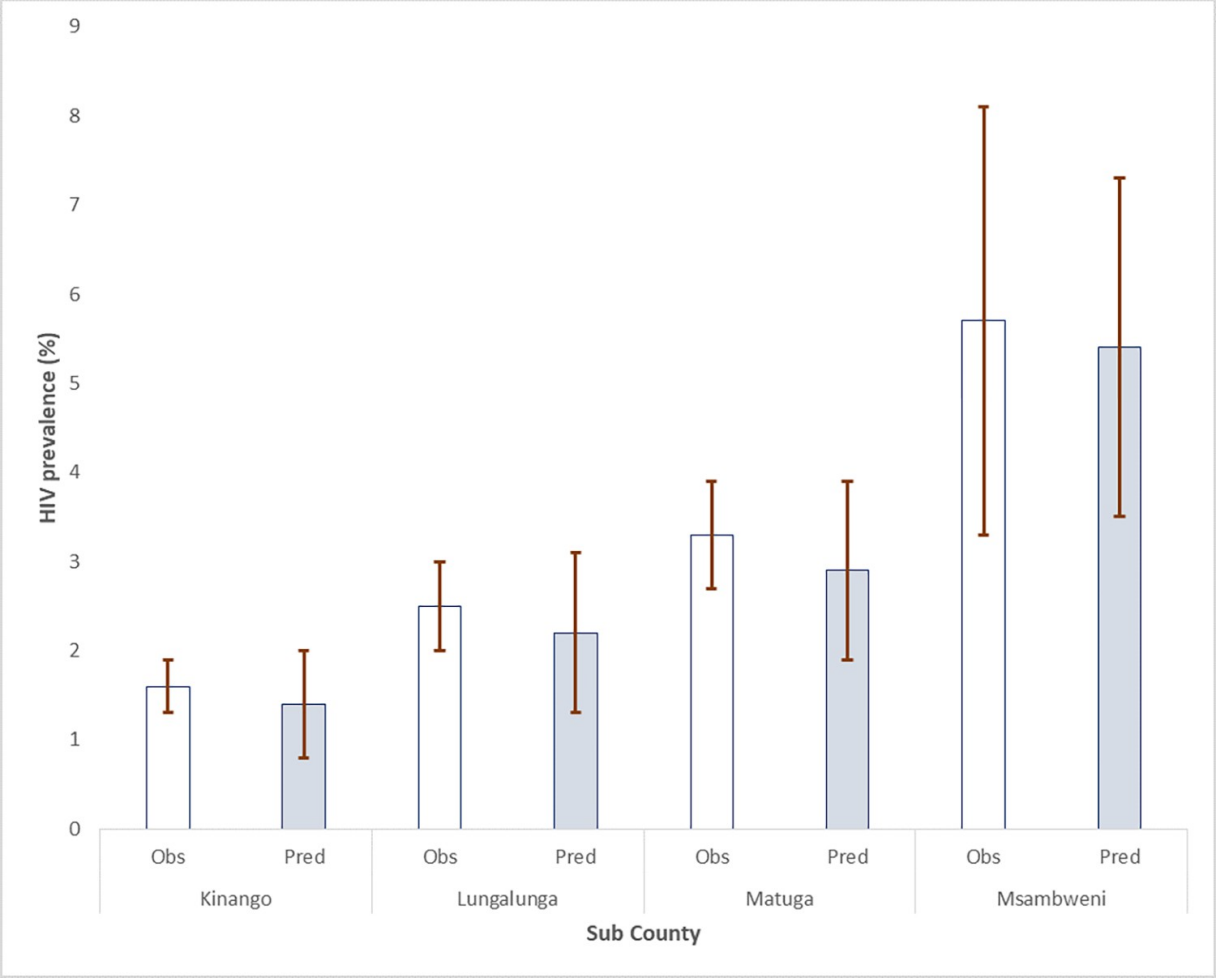
Observed and predicted HIV prevalence among all women by Sub County.

**Fig 4:**
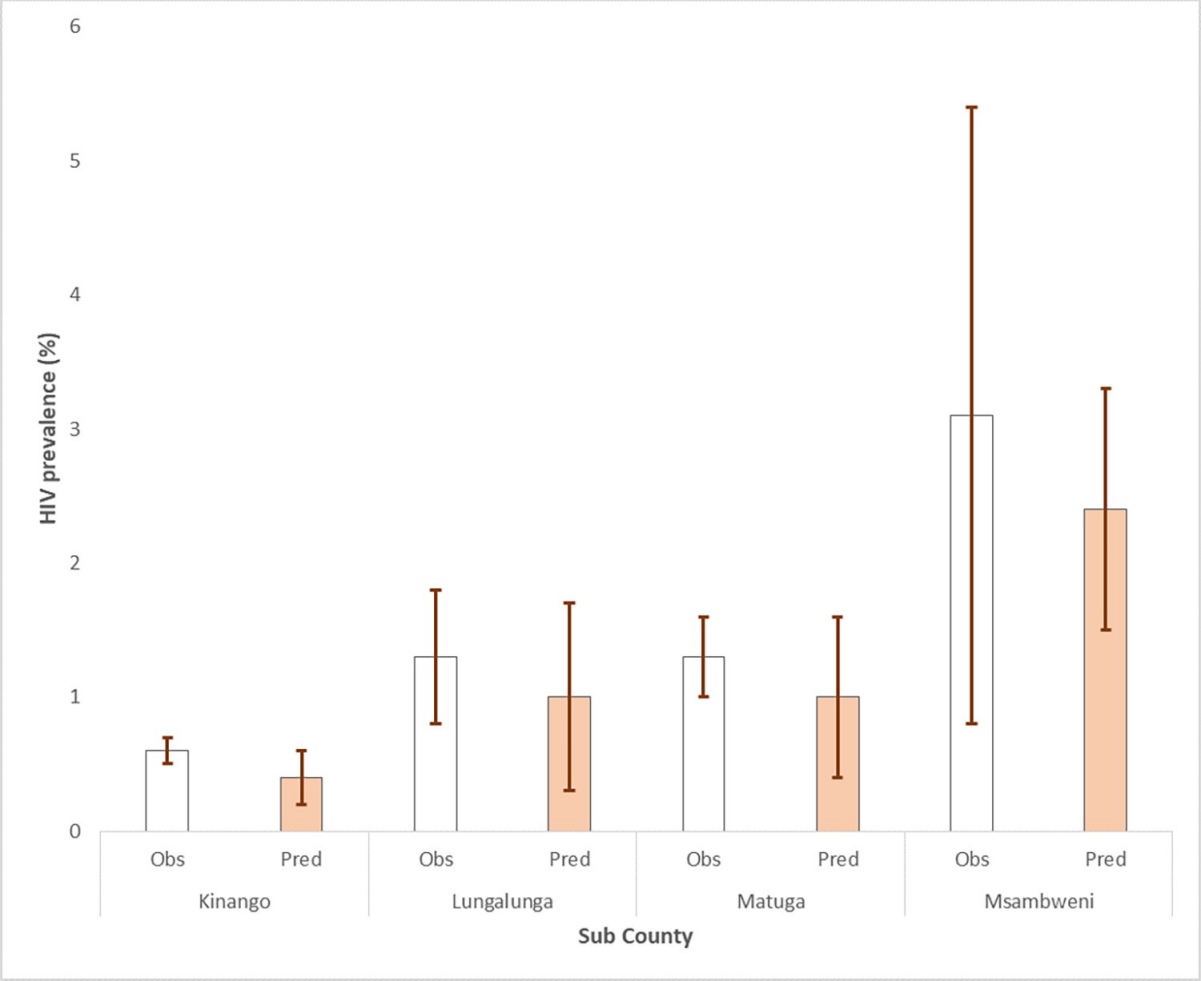
Observed and predicted HIV prevalence among newly diagnosed women by Sub County.

Figs 3 and 4 provide the means and confidence intervals of the observed and predicted prevalence.

Notably, Msambweni Sub County had the highest uptake of HIV testing with comparable levels of observed and predicted HIV prevalence.

## Discussion

The average HIV status ascertainment was at 91.3%, slightly above the UNAIDS 90% target [12]. The observed HIV prevalence was at 3.0%, but fluctuated across the study period, from a high of 3.7% in 2017 to a low of 2.5% in 2018. When considering all the women including those with a previously known status, there was no significant difference between the observed and predicted prevalence. A higher observed than predicted HIV prevalence would imply that women who attended ANC within that year may have been more at risk of HIV or the already HIV positive women were overrepresented. Additionally, this overrepresentation may be more profound if testing services are disrupted [11,13] as was the case in the 2017 protracted national industrial action by healthcare workers. Excluding known HIV positive women, the average HIV testing uptake was at 91.1%, slightly lower than if all women attending ANC were considered. The 2018 Kenya Population-based HIV Impact Assessment (KENPHIA) estimates the national and Kwale County HIV prevalence at 4.9% and 4.2% respectively [13]. The national prevalence among women of reproductive age (15 to 49 years) is estimated at 6.2% (95% CI 5.7–6.8%). While ANC data is useful in monitoring the HIV trends, it may not be representative of the general population. The lower HIV positivity in ANC as compared to the national population based surveys suggests that HIV positive women are underrepresented at the clinics. It further implies that there are HIV positive pregnant women within the community who are yet to access ANC services. The proportion of estimated pregnant women in Kwale County who attend first ANC visit ranged between 70% to 85% within the study period [2]. Underestimating prevalence at ANC may also be due to declining HIV testing among pregnant women who are more at risk of having HIV.

HIV exhibits geographical heterogeneity [14] thus contextual factors are key in understanding HIV burden estimates. In Kenya, HIV prevalence is higher in the rural (5.0%) as compared to urban areas (4.7%) [13]. Rural areas have been shown to be disproportionately affected by barriers of access to healthcare like illiteracy and socio-cultural practices like early marriages, that may negatively impact morbidity and mortality from communicable diseases [14]. This is contrary to the findings of this study that show no significant differences between prevalence in the relatively rural sub counties of Kinango and Lungalunga as compared to the more urban Matuga and Msambweni. Health facility level factors would also affect HIV status ascertainment and prevalence; Msambweni Sub County hosts the County referral hospital but despite having both the highest ascertainment and prevalence, the confidence intervals are too wide and not statistically significant

With adjustment for imperfect testing, the trend in predicted prevalence was fairly constant as was the case in a similar study in Malawi [14]. Among newly tested pregnant women, imperfect HIV status ascertainment led to an overestimation of the HIV prevalence across two sub counties (Kinango and Lungalunga) and in the year 2015 and 2016. These cases may suggest that women who were not offered HIV testing or who opted out of testing were likely to be HIV negative. In 2018, there were missed opportunities for identifying newly diagnosed cases despite having the highest ANC attendance and ascertainment of over 90%. This implies that in 2018, women who were likely to be HIV positive opted out of testing or were not offered a HIV test. ANC prevalence estimates is evidently dependent on who gets ascertained hence the need for 100% ascertainment [14].

Routine ANC data with optimal HIV testing has been shown to provide reliable data for monitoring the trends of HIV infection [15,16]. Contrary findings have however been observed citing data quality and test accuracy issues in routine ANC. A 2016 study in Kenya recommended that additional preparation was required before routine antenatal HIV testing data could supplement sentinel surveillance [8]. Other studies have shown that there can be low positive percent agreement of ANC test results compared to surveillance data and recommended an assessment of the impact of site-level differences on surveillance models to be used [17,18]. Our study provides sub national level estimates (both county and Sub County) of HIV among pregnant women, which are normally masked when national estimates are computed [17]. Additionally we included all the PMTCT sites within the County which is more representative than a sentinel surveillance model. This study thus provides baseline information for subsequent monitoring of the PMTCT program at county level. The study was however not without limitations. Only women who attended ANC were included in the analysis hence these estimates may not be representative of all women of reproductive age within the county i.e. it excluded non pregnant women and pregnant women not attending ANC. In many parts of sub-Saharan Africa, the HIV prevalence in women of reproductive age is higher than in men of the same age. In Kenya for instance, HIV prevalence of men aged 15–49 years is estimated at 2.7% (95% C1 2.4–3.1) as compared to 6.2% (95% CI 5.7–6.8) in women [13]. Thus, ANC HIV prevalence is not generalizable.These limitations may distort the estimates hence caveats to interpretation of ANC derived estimates must be observed and these estimates triangulated with other data sources for cross comparison.

## Conclusions

We conclude that routine PMTCT data can provide useful estimates on the burden of HIV and offers a feasible alternative to the ethical concerns raised with unlinked anonymous testing (UAT) model. Based on the study findings, sub optimal HIV status ascertainment at ANC would commonly overestimate the HIV prevalence among women attending ANC whereas the use of ANC routine data would underestimate the true HIV prevalence among women of reproductive age not attending ANC. There is thus need to address both community level (demand side) and health facility level (supply side) barriers to the uptake of ANC services if such estimates are to be reliable and more representative. We recommend advocacy at community level and outreach programs to ensure equitable access to ANC services, while addressing health facility level factors like adequate forecasting and quantification of HIV test kits as well as continued health worker trainings and supervisions to ensure compliance to national HIV PMTCT guidelines.
